# Is proactive frailty identification a good idea? A qualitative interview study

**DOI:** 10.3399/BJGP.2020.0178

**Published:** 2021-06-29

**Authors:** Ebrahim Mulla, Elizabeth Orton, Denise Kendrick

**Affiliations:** Division of Primary Care, University of Nottingham, Nottingham.; Division of Primary Care, University of Nottingham, Nottingham.; Division of Primary Care, University of Nottingham, Nottingham.

**Keywords:** frailty, general practitioners, primary care, qualitative research

## Abstract

**Background:**

In England, GPs are independent contractors working to a national contract. Since 2017, the contract requires GPs to use electronic tools to proactively identify moderate and severe frailty in people aged ≥65 years, and offer interventions to help those identified to stay well and maintain independent living. Little is currently known about GPs’ views of this contractual requirement.

**Aim:**

To explore GPs’ views of identifying frailty and offering interventions for those living with moderate or severe frailty.

**Design and setting:**

A sequential mixed-methods study of GPs in the East Midlands region of England — namely Derbyshire, Leicestershire, Lincolnshire, Nottinghamshire, and Northamptonshire — undertaken between January and May 2019.

**Method:**

GPs were made aware of the study via professional organisations’ newsletters and bulletins, GP email lists, and social media, and were invited to complete an online questionnaire. Responses were analysed using descriptive statistics and, based on those survey responses, GPs with a range of GP and practice characteristics, as well as views on identifying frailty, were selected to participate in a semi-structured telephone interview. Interview transcripts were analysed using framework analysis.

**Results:**

In total, 188 out of 3058 (6.1%) GPs responded to the survey and 18 GPs were interviewed. GPs were broadly supportive of identifying frailty, but felt risk-stratification tools lacked sensitivity and specificity, and wanted evidence showing clinical benefit. Frailty identification increased workload and was under-resourced, with limited time for, and access to, necessary interventions. GPs felt they lacked knowledge about frailty and more education was required to better understand it.

**Conclusion:**

Proactively identifying and responding to frailty in primary care requires GP education, highly sensitive and specific risk-stratification tools, better access to interventions to lessen the impact of frailty, and adequate resourcing to achieve potential clinical impact.

## INTRODUCTION

With there being an ageing population that is living longer with more comorbidities, frailty — defined as a state of increased vulnerability to adverse outcomes following stressor events^1^ — is a major concern to the NHS in England.^2^ Identifying frailty can help predict who is likely to have a fall,^3^ experience an unplanned admission to hospital or a care home,^4^ or die within the next year;^5^ as such, proactive identification of frailty in primary care offers an opportunity to delay or avert these negative outcomes.^6^ Historically, health systems have responded to people living with frailty in a reactive manner, usually following an acute presentation. However, proactively identifying older people with frailty provides an opportunity to intervene and alter the frailty trajectory, supported by the re-configuration of single-diseasefocused healthcare services into person-centred integrated healthcare systems.^4^^,^^7^^,^^8^

GPs in England are independent contractors providing holistic primary care services to a registered population. The services provided by GPs are specified in a nationally agreed General Medical Services (GMS) contract. An addition to this contract in 2017/2018 required general practices to use an evidence-based electronic frailty identification tool, such as the electronic Frailty Index, to risk stratify patients aged ≥65 years.^2^^,^^9^ For those stratified as moderately or severely frail, clinical assessment is required to confirm frailty status; those confirmed as severely frail require a clinical review. The clinical review should include: reviewing medications; a falls history, where clinically appropriate; and provision of relevant interventions.^2^

There is little published information on GPs’ views of proactively identifying and responding to frailty in older people. As such, the aims of this study were to:

- identify GPs’ characteristics and views on frailty identification in order to interview GPs with a diverse range of characteristics and views on the issues; and
- explore GPs’ views on identifying frailty and offering interventions for those living with moderate or severe frailty.

## METHOD

This sequential mixed-methods study included a survey of GPs to address the first aim and semi-structured interviews to address the second.

### Questionnaire survey

All currently practising GPs working in the East Midlands region — namely, Derbyshire, Leicestershire, Lincolnshire, Nottinghamshire, and Northamptonshire — were eligible to take part in the survey. A brief online questionnaire was developed (see Supplementary Box S1), which was informed by a review of the literature and peer reviewed by an expert advisory group from NHS England’s Older People and Person Centred Integrated Care team. The questionnaire comprised five questions on GP views on frailty identification, with responses to be marked on a five-point Likert scale ranging from ‘strongly agree’ to ‘strongly disagree’. There were nine further questions about GP characteristics (sex, age, and stage of career) and practice characteristics (electronic healthcare record [EHR] system, name of clinical commissioning group [CCG], practice size, whether it was a teaching practice, and whether it was a research practice). Responders were also asked to indicate whether they were interested in being interviewed following the survey.

**Table:** How this fits in

In England, the GP contract requires GPs to routinely identify older people with frailty and offer interventions to help them stay well and maintain independence. Little is known about GPs’ views of this requirement. This study found that most GPs are supportive of this, but wanted more education on frailty, improved tools to identify frailty, and evidence that identifying and responding to frailty makes a clinical difference. There was a lack of resources and time for frailty identification, along with a lack of access to interventions for older people living with frailty.

The questionnaire was piloted with eight GPs; minor amendments to wording to improve clarity were made subsequently. A written invitation to participate in the survey was circulated electronically through CCG e-newsletters, regional GP email lists, Royal College of General Practitioners faculty e-bulletins, NHS newsletters, and social-media networks covering the East Midlands region. The survey was open for 8 weeks between January and March 2019, and up to three reminders were made in total.

### Semi-structured qualitative interviews

This study adopted a pragmatic worldview,^10^ combining qualitative and quantitative methods sequentially^11^ to derive knowledge about topics covered by the research questions; the questions, in this case, were related to the implementation of systems to identify frailty. GPs who expressed interest in being interviewed were selected using maximum-variation sampling.^12^ The aim of the sampling was to purposely select GPs who differed from each other in terms of views on frailty identification reported in the survey, GP characteristics (age, sex, years as a GP, and role), and practice characteristics (EHR system, practice size, teaching practice, and research practice).

Interviews were conducted by one researcher, who did not know the interviewees, and the identity of the interviewees was not disclosed to the other researchers. The other two researchers have previously researched falls prevention and were aware that this may have influenced their view of the importance of frailty identification and referral for falls-prevention interventions. The interviewer and one other researcher acknowledged that their professional backgrounds will have likely engendered empathy with the GPs they interviewed, which may have influenced their subsequent coding of interviews. Interviewees were given information about the study aims in a participant information sheet and informed consent was obtained verbally prior to the interview. Interviews were conducted between March and May 2019; there was no financial incentive to take part.

Semi-structured telephone interviews^13^ were conducted using an interview schedule that was formulated from discussions within the research team, reviewed by an NHS England expert advisory group and piloted with three other GPs. The interview questions and prompts are shown in Supplementary Box S2. Interviews lasted 30–80 minutes, were conducted at a time convenient to GPs, and were audio-taped, anonymised, and transcribed verbatim by the interviewer and a university-approved transcription service. Data collection and thematic analysis were conducted in parallel, and interviews continued until data saturation was reached^14^ — that is, when no new themes emerged from the data.

### Data analysis

Frequencies and percentages of survey responses were calculated using an Excel database. Interview transcripts were imported to NVivo (version 11) to support coding and data organisation. Framework analysis^15^^–^^17^ was used to analyse interview transcripts. The analysis followed the stages described by Gale *et* *al*,^18^ using an inductive approach; this involved familiarisation with the interview by reading and re-reading the transcript, followed by reading the transcript line by line and applying a label (or code) to describe what the researcher has interpreted as important or anything they thought might be relevant (open coding).

The first two transcripts were independently coded by all three researchers; they then met to:

- discuss codes;
- agree the codes to use for further transcripts to ensure consistent coding; and
- group codes into categories or themes.

The identified themes formed the initial analytical framework; this was applied to the remaining transcripts, which were indexed with codes and themes. The framework was refined in an iterative process as further transcripts were analysed and additional codes added. Codes were compared within, and across, interviews, and themes and subthemes were agreed in meetings with all three authors. The authors searched for, but did not find, disconfirming cases^12^ that did not fit emerging themes.

Once all transcripts had been coded, data were entered into a matrix (charted) and summarised by theme and subtheme; illustrative quotations were included. Data interpretation occurred through meetings between all three authors, in which themes and subthemes were discussed, as were similarities, differences, and connections between themes.

## RESULTS

All in all, 188 GPs from 19 CCGs completed the survey. Characteristics of survey responders and their practices are outlined in Table 1. NHS England workforce data reported 3058 GPs registered on the Performers List in the 19 CCGs at the time of the survey,^19^ giving a response rate of 6.1%; a breakdown of the responses and data, by region, is given in Table 2.

**Table 1.** Characteristics of survey responders (*N* = 188) and their practices

**GP characteristics**	**Responders, *n* (%^a^)**
Sex	
Male	78 (42.9)
Female	104 (57.1)
Missing data	6

Age range, years	
21–29	7 (3.7)
30–39	69 (36.9)
40–49	48 (25.7)
50–59	50 (26.7)
>60	13 (7.0)
Missing data	1

Years in practice	
0–4	50 (27.0)
5–9	32 (17.3)
10–14	26 (14.1)
15–19	16 (8.6)
20–24	27 (14.6)
25–29	20 (10.8)
>30	14 (7.6)
Missing data	3

Job role	
GP partner	105 (57.1)
Salaried GP	41 (22.3)
Locum GP	17 (9.2)
Retainer GP	3 (1.6)
Other	18 (9.8)
Missing data	4

**Practice characteristics**	
Electronic healthcare record system	
SystemOne	150 (80.2)
EMIS	36 (19.3)
Vision	0 (0)
Other	1 (0.5)
Missing data	1

Number of patients registered at the general practice	
<3000	3 (1.6)
3000–5999	31 (17.0)
6000–8999	40 (22.0)
9000–11 999	24 (13.2)
>12 000	84 (46.2)
Missing data	6

Teaching practice^b^	155 (98.7)
Missing data	31

Research practice^c^	60 (38.2)
Missing data	31

a *Percentages have been calculated based on the total number of responders for each characteristic, not the total sample.*

b *Teaches undergraduate medical students or GPs in training.*

c *Takes part in research activities.*

**Table 2.** Survey responders by region out of non-missing data^a^

**Region**	**CCG**	**Responders, *n* (%)**	**Total GPs, *n*^b^**	**Total responders by region, *n***
Northamptonshire	NHS Nene CCG^c^	14 (7.7)	389	18
	NHS Corby CCG^c^	4 (2.2)	56	—

Leicestershire	NHS East Leicestershire and Rutland CCG	10 (5.5)	234	54
	NHS West Leicestershire CCG	24 (13.2)	260	—
	NHS Leicester City CCG	20 (11.0)	242	—

Nottinghamshire	NHS Nottingham City CCG^d^	24 (13.2)	287	67
	NHS Rushcliffe CCG^d^	10 (5.5)	92	—
	NHS Nottingham North and East CCG^d^	16 (8.8)	91	—
	NHS Nottingham West CCG^d^	8 (4.4)	82	—
	NHS Newark and Sherwood CCG^d^	6 (3.3)	83	—
	NHS Mansfield and Ashfield CCG^d^	3 (1.6)	98	—

Derbyshire	NHS Southern Derbyshire CCG^e^	15 (8.2)	737^f^	25
	NHS Erewash CCG^e^	8 (4.4)	—	—
	NHS North Derbyshire CCG^e^	1 (0.5)	—	—
	NHS Hardwick CCG^e^	1 (0.5)	—	—

Lincolnshire	NHS Lincolnshire West CCG^g^	11 (6.0)	122	18
	NHS Lincolnshire East CCG^g^	2 (1.1)	127	—
	NHS South Lincolnshire CCG^g^	1 (0.5)	87	—
	NHS South West Lincolnshire CCG^g^	4 (2.2)	71	—

Total		182	3058	182

a *Six GPs did not provide data on their CCG.*

b *Figures taken from NHS workforce data;^19^ includes GP partners, salaried GPs, GP retainers, and GP locums, but excludes GP registrars.*

c *These CCGs later combined to form NHS Northamptonshire CCG.*

d *These CCGs later combined to form NHS Nottingham and Nottinghamshire CCG.*

e *These CCGs later combined to form NHS Derby and Derbyshire CCG.*

f *Figures for GP headcount by CCG for Derbyshire area were not available.*

g *These CCGs later combined to form NHS Lincolnshire CCG. CCG = clinical commissioning group.*

A total of 35 GPs expressed interest in participating in an interview and 18 were subsequently interviewed. Interviewees’ GP and practice characteristics are outlined in Table 3; details by individual responder are given in Supplementary Table S1. Diversity was achieved in terms of GP characteristics (Table 3), most practice characteristics (Table 3), and four of the five questions on views about frailty (Table 4) — the exception being that all GPs interviewed agreed that the advantages of identifying frailty outweighed the disadvantages. Four main themes and subthemes emerged from the interviews (Box 1); these are explored below.

**Table 3.** Characteristics of interviewees (*N* = 18) and their practices

**GP characteristics**	**Interviewees, *n* (%)**
Sex	
Male	7 (38.9)
Female	11 (61.1)

Years in practice	
0–4	6 (33.3)
5–9	1 (5.6)
10–14	3 (16.7)
15–19	1 (5.6)
20–24	3 (16.7)
25–29	2 (11.1)
>30	2 (11.1)

Job role	
GP partner	9 (50.0)
Salaried GP	5 (27.8)
Locum GP	4 (22.2)
Retainer GP	0 (0.0)
Other	0 (0.0)

**Practice characteristics**	
Electronic healthcare record system	
SystemOne	16 (88.9)
EMIS	2 (11.1)
Vision	0 (0.0)
Other	0 (0.0)

Number of patients registered at the general practice	
<3000	0 (0.0)
3000–5999	5 (27.8)
6000–8999	4 (22.2)
9000–11 999	2 (11.1)
>12 000	7 (38.9)

Teaching practice^a^	
Yes	16 (88.9)
No	2 (11.1)

Research practice^b^	
Yes	8 (44.4)
No	10 (55.6)

a *Teaches undergraduate medical students or GPs in training.*

b *Takes part in research activities.*

**Table 4.** Responses from the survey (*N* = 188) and interview (*N* = 18)

**Survey statement and response**	**Survey responders, *n* (%)**	**Interviewees, *n* (%)**
The advantages of identifying and reviewing older people living with frailty in primary care outweigh the disadvantages		
Strongly agree	56 (29.8)	4 (22.2)
Agree	90 (47.9)	14 (77.8)
Neither agree nor disagree	28 (14.9)	0 (0.0)
Disagree	12 (6.4)	0 (0.0)
Strongly disagree	2 (1.1)	0 (0.0)

It has been easy to identify older people living with frailty in my practice		
Strongly agree	12 (6.4)	1 (5.6)
Agree	92 (48.9)	7 (38.9)
Neither agree nor disagree	45 (23.9)	3 (16.7)
Disagree	34 (18.1)	7 (38.9)
Strongly disagree	5 (2.7)	0 (0.0)

It has been easy to review older people living with frailty in my practice		
Strongly agree	3 (1.6)	0 (0.0)
Agree	34 (18.1)	4 (22.2)
Neither agree nor disagree	55 (29.3)	6 (33.3)
Disagree	72 (38.3)	6 (33.3)
Strongly disagree	24 (12.8)	2 (11.1)

Identifying and reviewing older people living with frailty in my practice has led to improvements in their care		
Strongly agree	12 (6.4)	0 (0.0)
Agree	71 (37.8)	8 (44.4)
Neither agree nor disagree	73 (38.8)	8 (44.4)
Disagree	21 (11.2)	2 (11.1)
Strongly disagree	11 (5.9)	0 (0.0)

Identifying and reviewing older people living with frailty is a good use of primary care resources		
Strongly agree	29 (15.4)	1 (5.6)
Agree	92 (48.9)	12 (66.7)
Neither agree nor disagree	41 (21.8)	4 (22.2)
Disagree	17 (9.0)	1 (5.6)
Strongly disagree	9 (4.8)	0 (0.0

**Box 1.** Themes and subthemes identified from the interviews

**Theme**	**Subthemes**
Beliefs about stratification and proactive identification of frailty	Universal stratification to risk profile patients
	Lack of supporting evidence
	Overreach
	Narratives

Stratification tools	Uncertainty about application of electronic tools
	Mixed impression of electronic tools
	Lack of sensitivity
	Lack of specificity
	Clinical confirmation of frailty

Managing complexity, resources, and models of care	Managing complexity well increases workload
	Insufficient time
	Trade-off between time and care
	Models of primary care
	Lack of resources in the community

Drivers of GP behaviour	Financial incentives
	Non-financial incentives
	Incomplete understanding of frailty

### Beliefs about stratification and proactive identification of frailty

#### Universal stratification to risk profile patients

Many GPs were positive about the idea of proactively identifying frailty, as highlighted by GP2 (male, locum, early career), who stated that:

*‘There is some worth* [to] *grading them* [patients] *on a traffic-light system.’*

Some GPs saw a benefit in intervening at an early stage to avert adverse outcomes:

*‘In principle, it is a really good idea … What I think it does and the reason I think it does have value is that it helps us identify cohorts of patients who are potentially at risk and who will benefit.’*

(GP13, male, partner, mid-career)

*‘Prevention is better than cure, so if you identify somebody that would be a good place to start.’*

(GP3, female, salaried, mid-career)

#### Lack of supporting evidence

Despite being supportive of the stratification approach, GPs had reservations, feeling that little could be done to influence frailty:

*‘We can identify and label people with diseases, but actually if there is not much you can do about it … I am not sure who is happier, or if anybody is.’*

(GP3, female, salaried, mid-career)

Many were keen to see evidence that proactive identification leads to improved patient care but were concerned this would become a tick-box or data-capture exercise:

*‘It would be interesting to see if this changed stuff — is it just going to be* [a] *tick-box exercise?’*

(GP10, female, locum, early career)

#### Overreach

There were concerns highlighted by one GP that proactively identifying these patients was an overreach of the role of primary care:

*‘It is a bit “nanny state” isn’t it? Most patients know when they need to see me. I don’t know whether it is right or not to impose ourselves on people, who are getting along quite happily.’*

(GP17, male, partner, late career)

#### Narratives

Another GP voiced concerns over the difficulty of stratifying the multifaceted concept of frailty using a number and proposed a narrative approach instead:

*‘I do wonder whether a narrative explanation would be better than fairly binary outcomes.’*

(GP2, male, locum, early career)

### Stratification tools

#### Uncertainty about application of electronic tools

Several GPs were unaware of how electronic frailty-stratification tools worked, which tool was being used by their practice, and how it was being applied:

*‘How is it being done? I’m not sure … I can’t tell you what algorithm they are applying. Our IT manager is applying it.’*

(GP4, female, partner, late career)

*‘I thought it was just something that appeared on SystemOne … I don’t know what the search criteria are.’*

(GP7, female, salaried, late career)

#### Mixed impression of electronic tools

There were mixed views on the overall helpfulness of the stratification tools:

*‘It is probably a bit of a blunt instrument.’*

(GP4, female, partner, late career)

*‘The frailty thing needs to be a lot more finely tuned than it currently is.’*

(GP7, female, salaried, late career)

*‘The algorithm is pretty kind of accurate.’*

(GP11, female, salaried, early career)

One GP felt that stratification tools were inherently flawed as they were based on multimorbidity, which is not the same as frailty:

*‘It is highly unlikely that a computerised calculation of all of the comorbidities of the patients has could accurately sum up their frailty.’*

(GP17, male, partner, late career)

#### Lack of sensitivity

Many GPs reported a mismatch between frailty scores and their perceptions of who was frail. Some patients did not score highly on the stratification tool and were classified into ‘mild’ or ‘not frail’ categories, despite the GP’s clinical judgement being that the patient was living with moderate or severe frailty. This particularly affected those without many long-term conditions and those living in care or nursing homes:

*‘There is definitely under-identification of people who are frail but don’t necessarily have lots of long-term conditions.’*

(GP4, female, partner, late career)

*‘It was something like fifty per cent of our care-home and nursing-home patients were not picked up by the electronic Frailty Index, which is obviously really significant because almost ninety-five per cent or more of that population is going to be frail.’*

(GP13, male, partner, mid-career)

#### Lack of specificity

GPs felt that some people scored highly on the stratification tool but were living with mild frailty or were not frail. This particularly affected patients living with many long-term conditions:

*‘Having undertaken quite a lot of reviews of patients who are tagged by the electronic Frailty Index as being severely frail, we found out that, actually, they are either not frail at all or moderately frail.’*

(GP14, male, partner, early career)

*‘It would throw up surprising people as having* [a] *high frailty index* [score] *… we looked at the top one hundred patients and I would think* [of] *at least twenty that we saw, there is no way they should be on this index.’*

(GP4, female, partner, late career)

#### Clinical confirmation of frailty

Clinical correlation to confirm frailty was performed by GPs through a variety of approaches including a rapid, intuitive, and informal diagnostic approach akin to ‘eye-balling the patient’, reviewing the patient record, and the GP’s previous knowledge of the patient:

*‘Obviously, eye-balling the patient and reviewing their notes properly and what is happening — no substitute for that really.’*

(GP11, female, salaried, early career)

Some GPs described using electronic frailty scores in isolation to diagnose frailty, unaware of the need for clinical correlation:

*‘*[The electronic tool] *probably has more of an idea than I have of* [who is moderately or severely frail] *… how else am I supposed to assess it, from a gut feeling?’*

(GP4, female, partner, late career)

### Managing complexity, resources, and models of care

#### Managing complexity well increases workload

Many GPs felt that a proactive approach to managing frailty increased workload through conducting clinical reviews that uncovered unmet needs that required further action:

*‘We’re generating more work for ourselves, because we are uncovering unmet need.’*

(GP13, male, partner, mid-career)

*‘… once you get them in the room and start asking them nice, friendly questions like you know how can we prevent you from falling and how can we optimise your care, then a whole load of worms come out of the cupboard don’t they.’*

(GP12, female, partner, late career)

#### Insufficient time

GPs unanimously found that managing patients with frailty resulted in extra workload and required extra GP time:

*‘When I do house visits for the severe*[ly] *frail* [older people] *, I go over all of the activities of daily living. When I went to do a routine review last week, I was there for an hour.’*

(GP4, female, partner, late career)

*‘Ten minutes is not enough for even* [an] *annual check-up … you should have at least twenty to thirty minutes* [to undertake a medication review and develop a care plan].*’*

(GP15, female, partner, early career)

#### Trade-off between time and care

Without this additional time, GPs felt they could not provide a high standard of care, as there was a direct trade-off between the two:

*‘They* [NHS England] *need to be aware that what they are asking takes a lot of time, especially if you want to do it well … If you were not particularly assiduous or committed to the whole process, you could tick a few boxes in five minutes and say “done it”.’*

(GP12, female, partner, late career)

#### Models of primary care

GPs described a variety of approaches for increasing time to deliver care to older people living with frailty. These included double appointments, follow-up appointments, or developing bespoke frailty clinics. However, they all reported difficulty in sustainably resourcing the extra time for this:

*‘We actually dedicated a clinic, so we actually saw twelve patients in a morning. They were all half-an-hour appointments. You felt like you were giving really good care, but the problem was it wasn’t sustainable because we just couldn’t keep on giving that amount of time to that activity.’*

(GP4, female, partner, late career)

*‘I suggest that people try and have double appointments, but it is often not practical because we are just trying to meet the base level of needs.’*

(GP9, male, salaried, early career)

#### Lack of resources in the community

GPs felt that community services were not able to meet the needs of older people living with frailty as they were under-resourced. Many GPs felt that, in order to be useful, these services also needed to be responsive to urgent need:

*‘What is available is an under-resourced physio and OT* [occupational therapy] *team. If we are going to kick people out into the community, we need the resources to do that and get them in* [to community services] *quickly.’*

(GP4, female, partner, late career)

*‘What is frustrating is that you identify all sorts of needs, but know the service is not going to be able to deal with them.’*

(GP5, female, locum, late career)

*‘The key is to have reactive services that are able to adapt to the unpredictable needs of the day quickly.’*

(GP14, male, partner, early career)

### Drivers of GP behaviour

#### Financial incentives

Many GPs felt that financial considerations were important, and it was imperative to receive appropriate funding to carry out the additional work. This included funding the time of GPs, time of other healthcare staff, and contributing to overhead costs:

*‘It is important to get paid for the time.’*

(GP1, male, locum, early career)

*‘How it is funded in the contract doesn’t really adequately take into consideration the time you put in with these patients.’*

(GP13, male, partner, mid-career)

*‘If you are running a business, you have to make ends meet. If you have a contract that says that you get paid X number of pounds per patient to deliver this service, you are going to do that* [deliver the service] *because general practice can’t run without the funding.’*

(GP14, male, partner, early career)

Some GPs viewed funded work as compulsory and non-funded work as of lower priority, even if it was clinically beneficial:

*‘Because we are not paid to do moderate-frailty assessments, I am much more inclined to not do them if I haven’t got time.’*

(GP4, female, partner, late career)

#### Non-financial incentives

One GP commented on the fact that financial incentives appeared to be the only type of incentives that are considered:

*‘You get the impression* [that the health secretary] *and NHS England know no other rules in incentivising individuals, who are professionals other than QOF* [Quality and Outcomes Framework; a financially incentivised part of the GMS contract] *and funding.’*

(GP2, male, locum, early career)

#### Difficulties measuring complex activity

Other GPs felt that managing frailty has been standard professional practice for GPs. The difficulty in directly measuring this, however, means it has neither been recognised by the wider health service, nor directly funded by commissioners:

*‘*[Frailty] *is one of the many things that I think GPs do quite well, but isn’t really acknowledged by the NHS or secondary care.’*

(GP2, male, locum, early career)

*‘What happens is that, in hospitals, you do activity and you get paid and measured on the activity. What we do is actually preventing activity and so it is harder to measure* […] *you can measure the number of care plans and admissions, but those are really blunt tools because, the reality is that, what we do is make differences to lives and that is a harder thing to measure.’*

(GP18, male, partner, late career)

#### Incomplete understanding of frailty

As highlighted by GP1 (male, locum, early career), several GPs recognised that frailty *‘is a term that is often used, but not really understood’.* This hampered GPs’ ability to identify and manage older people living with frailty; many felt their knowledge about frailty was incomplete and remarked that they did not know how to differentiate between the different grades:

*‘I have been a GP thirty-five years-plus and these are new terms to us for our understanding … who is severely frail and who is moderately frail.’*

(GP4, female, partner, late career)

*‘I don’t really know what the difference is. I would say that severe frailty is quite evident, but I wouldn’t know how to go about grading it.’*

(GP9, male, salaried, early career)

It was suggested that *‘We have to re-educate everybody about it’* (GP15, female, partner, early career) and, although it was felt that the value of experiential learning should be appreciated, suggestions were made to improve knowledge through structured education in undergraduate and postgraduate curricula, postgraduate qualifications, and protected learning time for GPs:

*‘GP’s pick it up and they get quite good at it, but they are not necessarily trained, it is more experiential learning on the job, rather than addressed at the front end of their career … It is something that needs to be a bit more structured in the undergraduate and postgraduate curriculums* [ *sic*] *… There are postgraduate qualifications.’*

(GP2, male, locum, early career)

*‘PLTs* [protected learning times] *would be quite good; teaching on it would be nice.’*

(GP16, male, salaried, early career)

## DISCUSSION

### Summary

This study found that GPs were broadly supportive of identifying frailty, but felt risk-stratification tools lacked sensitivity and specificity. Clinical correlation to confirm frailty was not universally performed. Some GPs were sceptical of the clinical impact of identifying frailty and the subsequent patient reviews for those living with severe frailty.

Frailty identification increased workload and identified unmet need. It was perceived to be under-resourced, with GPs having limited time to undertake clinical and medication reviews, as well as a lack of access to necessary community-based interventions. Many GPs felt that managing older people living with frailty is a core part of the GP’s job, but said they lacked knowledge about frailty and more education about it was required.

### Strengths and limitations

There are several strengths to this study, the first being that there has been little research to date^20^ exploring GPs’ perceptions of the impact of the frailty requirement introduced in the 2017/2018 GMS contract in England. The qualitative approach used allowed for an in-depth exploration of GPs’ views, and using the telephone to undertake the interviews was sympathetic to the schedules of the time-pressed GPs; it allowed for more flexibility and may have enabled GPs to feel less inhibited in disclosing their views.^21^ The authors recognised that, as practising clinicians, data collection and analysis might be influenced by their opinions and clinical experience, so reflected on this during data analysis and interpretation. In addition, the interview schedule was informed by expert opinion from an NHS England advisory group. A sample of transcripts was coded by all three authors, who also agreed emerging themes in an iterative process.

The purpose of the survey was to identify GPs to be interviewed. The survey results have been presented to show the range of characteristics and views among responders from whom the interview sample was chosen. The survey findings have not been interpreted further as, due to the low response rate, the views may not be representative of those of the wider population of GPs.

Diversity of interviewees was achieved in terms of GP and most practice characteristics, and for four of the five survey questions on views about frailty. However, all interviewees agreed that the advantages of identifying frailty outweighed the disadvantages; only 6.4% of survey responders disagreed with this statement, limiting the authors’ ability to select GPs with these views.

Despite using multiple methods to invite GPs to complete the survey, the low response rate raises the possibility that responders held differing views from non-responders. As with all survey and interview studies, social desirability bias may have occurred, with responders replying in a way they considered would be viewed favourably. Although interviewees did express negative views about various aspects of identifying and responding to frailty, it is possible that the findings represent more positive views about frailty identification than those of the wider GP community.

### Comparison with existing literature

It was found that GPs are open and keen to think about, and address, frailty, which supports the findings of previous studies.^20^^,^^22^ The findings presented here echo past concerns that population frailty stratification is unsupported by evidence and could turn into a ‘bureaucratic exercise’,^22^ and that frailty, itself, is oversimplification of complexity.^23^ The benefit of the additional work required to both identify and manage frailty needs to be justified, as it is happening against a backdrop of increasing workload, a falling number of GPs, and recognition of the need to increase GP consultation time.^24^

Electronic frailty stratification tools are quick and simple to use, and their construct validity for frailty identification has been previously demonstrated.^25^ However, similar to previous studies — such as that by Alharbi *et al*^20^ — the study presented here found that GPs perceive that the tools lack sensitivity and specificity, which has a negative impact on their real-world utility. Many GPs felt they knew which patients were frail, without the need for a formal identification test; this reflects previous study findings that GPs commonly employ their intuition to identify older people living with frailty.^26^ Previous findings that GPs lack knowledge about frailty^27^ are supported by those presented here; GPs interviewed by the authors did, however, suggest ways to ameliorate this.

The National Institute for Health and Care Excellence has previously suggested that the proactive approach is cost neutral, with associated expenses (training, treatment optimisation, longer appointments, and so on) being offset by other factors (such as fewer unnecessary appointments, prescriptions, and unplanned admissions).^28^ The findings of the study presented here, which are consistent with previous research,^20^ suggest this is not reflected in the experiences of those GPs who are implementing proactive frailty identification, as they reported additional primary care resource requirements, increased demand for community services (which may or may not exist), and scepticism about the clinical benefit.

### Implications for research and practice

Although a stratification and identification approach to frailty is largely supported by GPs, research is needed to demonstrate the clinical benefit before there is more universal acceptance of frailty identification by primary care professionals. Further research is also required into the accuracy of stratification tools, with a focus on characterising and reducing the number of older people being misidentified to ensure utility of the tools for clinical practice.

Although the contractual requirement for identifying and assessing people living with frailty has raised awareness and increased activity in this area, it has added to the primary care workload without being adequately resourced; this is unlikely to be a sustainable model in the long term. Providing payment only for intervening with patients who are living with severe frailty acts as a disincentive for addressing moderate frailty, despite evidence of, at the least, some effective interventions across the frailty spectrum.^29^ There are always competing demands on the time of primary care professionals; nevertheless, the findings presented here indicate that more education and training is required for clinicians to successfully identify and respond to frailty.^30^

Inadequate access to community-based services — for example, falls-prevention programmes or comprehensive geriatric assessments — means that needs identified during frailty reviews cannot always be met. Unless these issues are addressed, it is likely that any clinical benefit arising from the contractual requirement will be limited.
